# *Plac8* is required for White Adipocyte Differentiation in vitro and Cell Number Control in vivo

**DOI:** 10.1371/journal.pone.0048767

**Published:** 2012-11-14

**Authors:** Maria Jimenez-Preitner, Xavier Berney, Bernard Thorens

**Affiliations:** Center for Integrative Genomics, University of Lausanne, Lausanne, Switzerland; Universita Magna-Graecia di Catanzaro, Italy

## Abstract

Plac8 belongs to an evolutionary conserved family of proteins, mostly abundant in plants where they control fruit weight through regulation of cell number. In mice, *Plac8* is expressed both in white and brown adipose tissues and we previously showed that *Plac8^−/−^* mice develop late-onset obesity, with abnormal brown fat differentiation and reduced thermogenic capacity. We also showed that in brown adipocytes, *Plac8* is an upstream regulator of *C/EBPβ* expression. Here, we first assessed the role of *Plac8* in white adipogenesis in vitro. We show that *Plac8* is induced early after induction of 3T3-L1 adipocytes differentiation, a process that is prevented by *Plac8* knockdown; similarly, embryonic fibroblasts obtained from *Plac8* knockout mice failed to form adipocytes upon stimulation of differentiation. Knockdown of *Plac8* in 3T3-L1 was associated with reduced expression of *C/EBPβ*, *Krox20*, and *Klf4*, early regulators of the white adipogenic program, and we show that *Plac8* could transactivate the *C/EBPβ* promoter. In vivo, we show that absence of *Plac8* led to increased white fat mass with enlarged adipocytes but reduced total number of adipocytes. Finally, even though *Plac8^−/−^* mice showed impaired thermogenesis due to brown fat dysfunction, this was not associated with changes in glycemia or plasma free fatty acid and triglyceride levels. Collectively, these data indicate that *Plac8* is an upstream regulator of *C/EBPβ* required for adipogenesis in vitro. However, in vivo, *Plac8* is dispensable for the differentiation of white adipocytes with preserved fat storage capacity but is required for normal fat cell number regulation.

## Introduction

Adipogenesis is the process by which fibroblastic-like preadipocytes differentiate into adipocytes capable of storing fat in the form of triglycerides [1], [2]. In vivo, white adipocytes store triglycerides in a single large lipid droplet from which free fatty acids can be released during the fasted state and secreted in the blood to provide metabolic energy to other tissues, such as muscle and liver. Imbalance between fat storage and release by adipocytes may lead to gain or loss of body weight. In obesity, excess fat storage and adipocyte enlargement are often associated with local inflammation and insulin resistance, production of cytokines, which can propagate insulin resistance to other tissues, and exaggerated lipolysis causing storage of fat in liver, muscles, or pancreatic beta-cells [3], [4], [5].

Understanding the molecular pathways controlling adipocytes differentiation from precursor cells is therefore important as this knowledge may help control adipocyte number and fat mass. A large body of research has identified a transcriptional cascade regulating white and brown fat differentiation. Common mechanisms controlling the differentiation of both types of fat tissues include activation of the CAAT/Enhancer Binding Protein ß (C/EBPβ), which activates C/EBPα and C/EBPδ; these transcription factors then stimulate expression of peroxisome proliferator-activated receptor γ (PPARγ) [1], [2], [6]. In white adipocytes, the transcription factors Krox20 and Klf4 are upstream regulators of C/EBPβ [7], [8] whereas brown fat-specific differentiation requires the interaction of C/EBPβ with the zinc finger-containing protein PRDM16, which leads to adipogenic development through induction of PPARγ and mitochondrial biogenesis through subsequent activation of peroxisome proliferator-activated receptor γ-coactivator 1α (PGC-1α). Whereas genetic inactivation of PPARγ prevents adipocyte development, inactivation of C/EBPβ is still compatible with both white and brown adipose tissue development but prevents normal function of brown fat [6], [9].

In a recent study we identified *Plac8*, also called *onzin*, as a novel upstream regulator of the brown adipocyte differentiation program. Plac8, a 124 amino acid long protein, shows structural similarity with the product of the *fruit weight 2.2* (*fw2.2*) gene, which controls fruit weight in tomato, through a regulation of cell number. In plants there are many *fw2.2*-related genes whereas in mammals *Plac8* appears to be the unique member of this family [10]. Plac8 contains a cysteine-rich sequence located between amino acids 23–66 (the Plac8 domain). We showed in a recent report that upon induction of brown preadipocyte differentiation Plac8 transiently interacts with C/EBPβ. The Plac8/C/EBPβ complex then binds to tandem C/EBPβ binding sites present on the *C/EBPβ* gene promoter to induce this gene transcription. Interaction of Plac8 with C/EBPβ requires the initial part of the cysteine-rich region (a.a. 28–38) and Plac8 deletion mutants lacking this sequence can no longer rescue the differentiation of *Plac8^−/−^* brown preadipocytes. *Plac8^−/−^* mice have abnormal brown adipocytes characterized by a single large lipid droplet and impaired thermogenesis leading to lower body temperature and cold intolerance [11]. Over time, these mice develop obesity for reasons that may be related to defects in thermogenesis and, because Plac8 is also expressed in white adipocyte, to a defect in this tissue homeostasis.

Therefore, here, we investigated the impact of *Plac8* inactivation on white adipocyte differentiation in vitro and on white adipose tissue in *Plac8^−/−^* mice. We show that *Plac8* is required for in vitro adipogenesis through a regulation of *C/EBPβ* expression and in vivo it is dispensable for white fat depots production but is required to properly control white fat mass.

## Material and Methods

### Mice

*Plac8^−/−^* mice on a pure C57Bl/6 background were obtained from Dr Koller's laboratory (University of North Carolina) [12]. C57Bl/6 and *Plac8^−/−^* mice were crossed to obtain *Plac8^+/−^*, and then *Plac8^+/−^* mice were crossed to obtain *Plac8^+/+^* ^,^, *Plac8^+/−^* and *Plac8^−/−^* littermates. Mice were maintained at 24°C on a 12 h/12 h light/dark cycle (7:00–19:00 h), with free access to water and standard chow diet (n° 3436 from Provimi Kliba AG, Kaiseraugst, Switzerland) and were used when 24–29 weeks old when the obesity was established. All animal studies were approved by the Veterinary Office of Canton de Vaud, Switzerland.

### Glucose, free fatty acids and triglycerides measurements

Blood samples were obtained from retroorbital bleeding in fed state or after a 16-hour overnight fasting in 24-weeks-old male mice. Analyses were performed using the Roche/Hitachi Robot 902 (Roche Diagnostics) and Roche kits (Roche, Basel, Switzerland).

### Histology and morphometry

Visceral and subcutaneous adipose tissues from 29-weeks-old male mice were embedded in paraffin and 5 µm sections were prepared for hematoxylin and eosin staining. Hematoxylin-eosin images from subcutaneous and visceral adipose tissues were used for morphometric analysis using the AxioVision 4.0 software (Zeiss, Oberkochen, Germany). 100 cells were measured using the software per animals.

### RNA extraction and real-time PCR

Total RNA was prepared from white adipose tissues from 29-weeks-old male mice or cultured cells using peqGOLD TriFast reagent according to the manufacturer's instructions (Axonlab, Baden, Switzerland). First strand cDNA was synthesized from 0.5 µg of total RNA using random primers (Promega, Mannheim, Germany) and Superscript II RNase H- (Invitrogen, Carlsbad, CA). Real time PCR was performed using Power SYBR Green Master Mix (Applied Biosystem, Foster City, CA, USA). Specific mouse primers for each gene were used and are described in Table S1. All reactions were normalized to cyclophilin levels and performed in triplicate.

### Cells protein extraction and Western blot analysis

Cells were lysed using a RIPA buffer containing SDS 0.1% and protease inhibitors (Roche, Basel, Switzerland). Western blot analysis was performed as previously described [13]. 20 mg of total protein were loaded on a 12% SDS-PAGE for each sample. Rabbit anti-Plac8 antibody was obtained from Dr. Koller's laboratory [12] and used at a dilution of 1/2500, rabbit anti-α-actin (Sigma, A2066, St-Louis, MO) and peroxidase conjugated anti-rabbit IgG (Amersham, NA934V, Glattbrugg, Switzerland) were used as secondary antibodies at a dilution of 1/1000. SuperSignal^R^ West Pico Stable Peroxide solution (Pierce, 1859674, Rockford, IL) was used for chemiluminescent detection.

### Generation of retroviral constructs and retroviral transductions

Plac8 cDNA was inserted in pMSCV retroviral vectors (Clontech, Mountain View, CA) with puromycin or hygromycin selection markers. C/EBPβ pMSCV retroviral construct was kindly provided by Dr. Kivanc Birsoy (Massachussets Institute of Technology, MA). Viral constructs were transfected using the calcium-phosphate protocol previously described [14] into Phoenix packaging cells (Pr. G. Nolan, Standford) along with constructs encoding gag-pol and the VSV-G protein and supernatants were harvested after 48 h in presence of 3 mM of trichostatin A (Sigma, St-Louis, MO) and either used immediately or snap frozen and stored at −80°C for later use. Viral supernatants were added to the cells for 6 hours in the presence of Polybrene (8 mg/ml, Sigma, St-Louis, MO), and then diluted twice with fresh medium. Next day, the viral supernatant was removed and replaced by fresh medium. Each transduction experiment was repeated three times.

### MEF preparation and Cell culture

Mouse embryonic fibroblasts were isolated from *Plac8^+/+^*, *Plac8^+/−^* and *Plac8^−/−^* day 14 embryos using the following protocol. Whole embryos was sliced in small pieces and incubated at 37°C for 1 hours in ES trypsin buffer (2.5 mg trypsin, 0.4 mg EDTA, 7 g NaCl, 0.3 mg Na_2_HP0_4_, 0.24 mg KH_2_PO_4_, 0.37 mg KCl, 1 mg dextrose, 3 mg Tris and 1 ml Difco phenol Red, pH 7.6). Then add 4 ml of DMEM (Gibco, Carlsbad, CA) with 10% FBS (Gibco, Carlsbad, CA,) and mixed the digested embryos. Add another 15 ml of medium and incubate the dispersed cells in a T75 flask. After 48 hours, split the cells to two T175 flasks. The primary mouse embryonic fibroblast can be used until passage 8–9. MEF and 3T3-L1 cell line (ATCC, http://www.lgcstandards-atcc.org/) were cultured in DMEM supplemented with FBS at 5% CO_2_. Cells were allowed to grow to confluence in either 100-mm or 60-mm dishes in DMEM with 10% FBS. Once confluence was reached, cells were exposed to differentiation medium containing dexamethasone (1 mM), insulin (5 mg/ml), isobutylmethylxanthine (0.5 mM). After 2 days cells were maintained in medium containing insulin (5 mg/ml). Medium was changed every other day. Full differentiation was achieved after 7 days.

### Oil red O staining

After 7 to 10 days of differentiation, cells were washed once in PBS and fixed with formaldehyde (Formalde-fresh; Fisher, Waltham, MA) for 15 minutes. The staining solution was prepared by dissolving 0.5 g oil-red-O (Sigma, St-Louis, MO) in 100 ml of isopropanol; 60 ml of this solution was mixed with 40 ml of distilled water. After 1 hour at room temperature the staining solution was filtered and added to dishes for 4 hours. The staining solution was then removed and cells were washed twice with distilled water.

### Trans-activation assays

The 3 kb *C/EPBβ* promoter in pGL3 plasmid was a gift from Dr Kivanc Birsoy (Boston, MA). The pMSCV retroviral plasmid for Plac8 was used as expression vector. The 1.3 kb *Krox20* promoter was obtained from Addgene (http://www.addgene.org/). 3T3-L1 preadipocytes cell line were transfected at 80% confluence using Lipofectamine™ LTX Reagent (Invitrogen, Carlsbad, CA) as per the manufacturer's protocol. A ß-galactosidase expression vector was co-transfected into cells as a control for transfection efficiency. Luciferase and ß-galactosidase activity were assessed 2 days after differentiation using the Galacto-Star luciferase reporter assay (Roche, Basel, Switzerland) following the manufacturer's instructions. Transfections were performed in triplicate and repeated three times. Statistics were performed using the Student *t*-test.

### shRNA constructs

shRNAs were constructed using the pSIREN RetroQ vector (Clontech, Mountain View, CA). Two different target sequences for Plac8 were designed by querying the Whitehead siRNA algorithm (http://jura.wi.mit.edu/bioc/siRNAext/) as well as the siRNA designer software from Clontech (http://bioinfo.clontech.com/rnaidesigner/). The sequences chosen for *Plac8* were TCGTGACTCAACCTGGATT and ACGGCATTCCTGGATCTAT. Retroviral production, transduction of 3T3-L1 cell line and differentiation protocols were as described above. Oil-red-O staining was performed at day 7. Each experiment was repeated three times.

### DNA content

VAT and SCAT tissues from 29-weeks-old male mice were homogenized with a Polytron in 1.5 ml PBS containing protease inhibitors (Roche, Basel, Switzerland), followed by a centrifugation at 11000 RPM for 5 minutes. The supernatant was placed in another tube for the DNA content assay. DNA content was measured using the Hoechst Method [15].

### Statistical Analysis

Statistical analyses were performed using the Graphpad Prism 4.0 software (Graphpad Software Inc.). Comparisons between groups were performed using the Student *t*-test.

## Results

**Figure 1:**
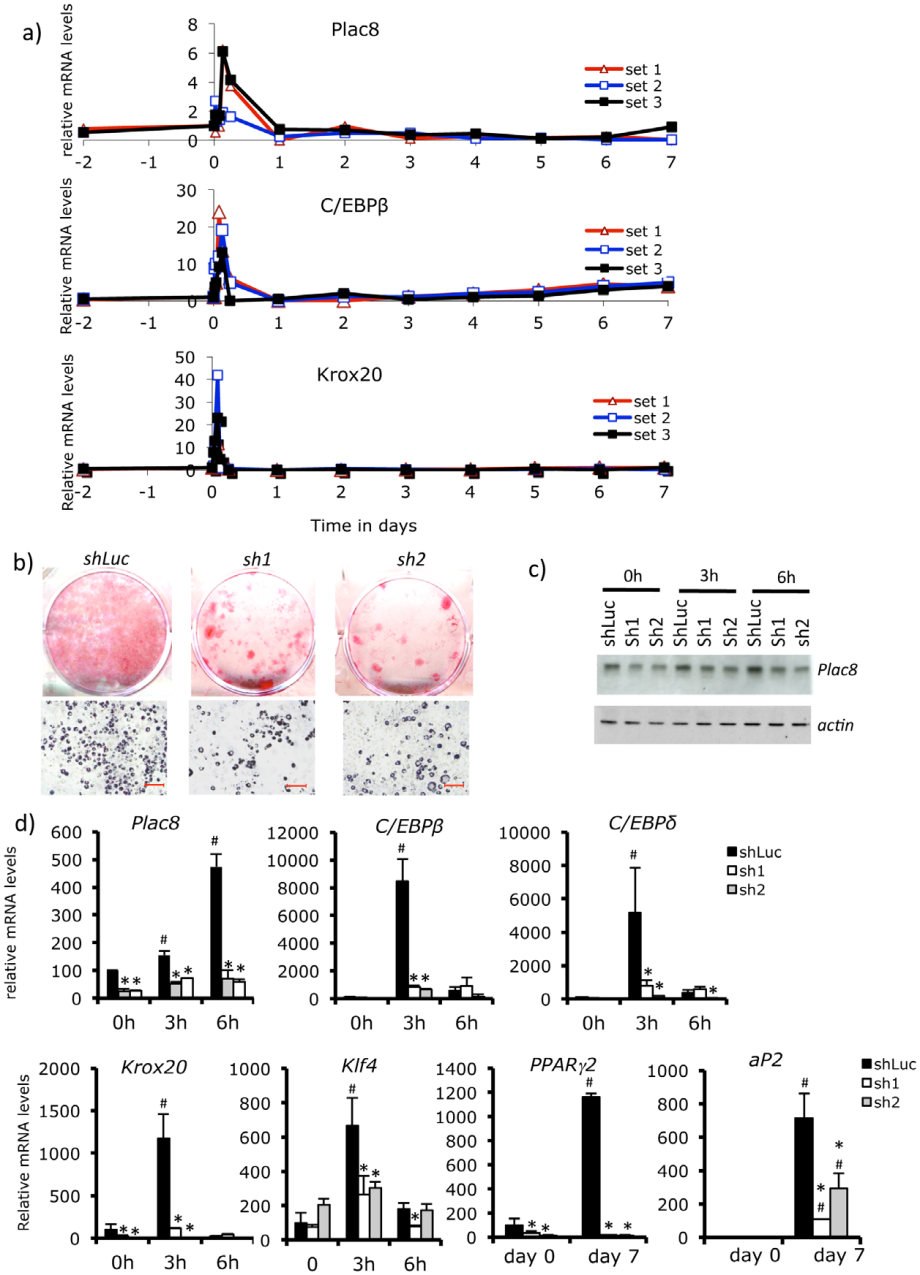
*Plac8* knockdown in 3T3-L1 decreases adipogenesis. **A**, Time-course (0, 3, and 6 h, 1 to 7 days) of *Plac8*, *C/EBPβ* and *Krox20* mRNA expression during 3T3-L1 cells differentiation. Three independent experiments are shown. **B**, Oil red O staining of 3T3-L1 transduced with two different *Plac8* (sh1 and sh2 and a control (shLuc) shRNAs at 7 days after induction of differentiation. Upper row: culture dishes; lower row: photomicrographs of the cells (scale bars = 100 µm). **C**, Western blot analysis of Plac8 protein in shRNA-transduced 3T3-L1 cells at day 0 and 3 and 6 hours after induction of differentiation. Actin served as loading control **D**, mRNA levels of *Plac8* and of the transcriptional regulators *C/EBPβ*, *C/EBPδ*, *Krox20*, *Klf4 and PPARγ2* and of the general adipogenic gene *aP2* at day 0 and 7 of differentiation. One representative experiment out of three is shown. Values are means ± SD, n = 3.,*p<0.05 *vs.* shLuc, #p<0.05 *vs.* shLuc at day 0.

To evaluate the role of *Plac8* in white adipogenesis we first assessed the pattern of *Plac8* expression during differentiation of 3T3-L1 adipocytes. Figure 1A shows that upon induction of differentiation, there was a rapid and transient induction of *Plac8* expression, which corresponded closely to the pattern of expression of *C/EBPβ* and *Krox20*, two transcription factors required early during white fat differentiation. ShRNA-mediated down-expression of *Plac8* reduced 3T3-L1 differentiation as determined by Oil Red O (ORO) staining (Figure 1B); this was associated with reduced expression of Plac8 protein and mRNA levels, as well as a reduced expression of *C/EBPβ*, *Klf4*, *C/EBPδ*, *Krox20*, *Pparγ2* and *Ap2* (Figure 1C,D). Expression of other adipocyte-specific genes, *Ebf1*, *Gata3*, *Nur77* and *cyclinD1* were, however, not affected by *Plac8* knockdown (Figure S1). With shRNA-mediated knockdown of *Plac8*, addition of rosiglitazone, a potent PPARγ agonist, also failed to restore the full differentiation of 3T3-L1 cells, as shown by ORO staining and gene expression analysis (Figure S2).

**Figure 2:**
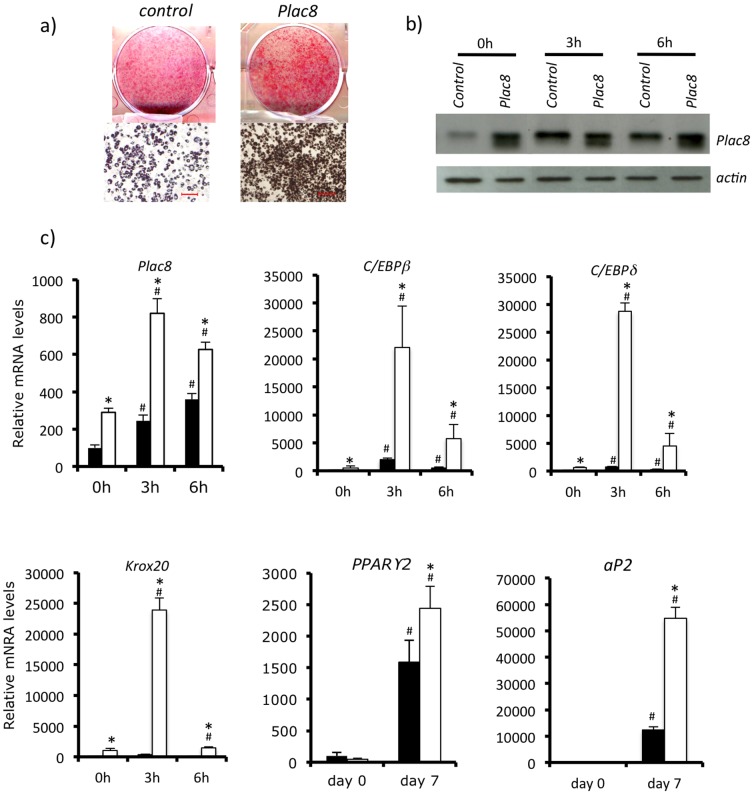
*Plac8* overexpression in 3T3-L1 enhances adipogenesis. **A**, 3T3-L1 cells were transduced with *Plac8* or control retroviruses. Oil red O staining was performed at 7 days after induction of differentiation. Upper row: culture dishes; lower row: photomicrographs of the cells (scale bars = 100 µm). **B**, Western blot analysis of Plac8 in shRNA transduced 3T3-L1 cells at day 0 and 3 and 6 hours after induction of differentiation. Actin served as loading control **C**, mRNA levels of *Plac8* and of the transcriptional regulators *C/EBPβ*, *C/EBPδ*, *Krox20 and PPARγ2* and of the general adipogenic gene *aP2* at days 0 and 7 of differentiation. One representative experiment out of three is shown. Values are means ± SD, n = 3.,*p<0.05 *vs.* shLuc, #p<0.05 *vs.* shLuc at day 0.

To determine whether overexpression of *Plac8* could increase differentiation, 3T3-L1 preadipocytes were transduced with *Plac8*-expressing retroviruses (Figure 2). This led to increased Plac8 expression and differentiation of 3T3-L1 adipocytes as revealed by ORO staining and by expression of *C/EBPβ*, *C/EBPδ*, *Krox20*, *PPARγ2* and *aP2* (Figure 2A, B, C).

**Figure 3:**
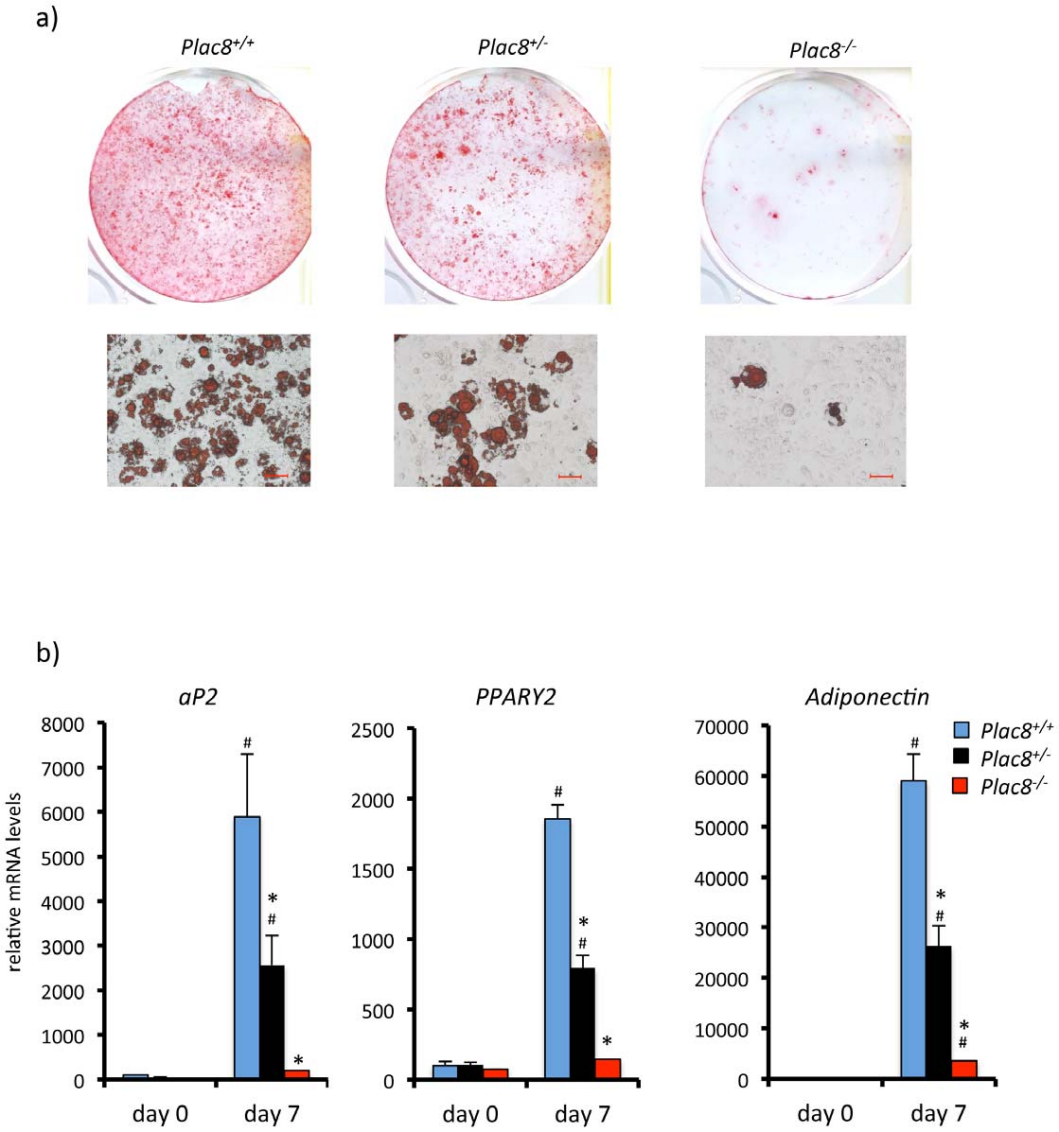
Absence of *Plac8* prevents adipogenic differentiation of primary mouse embryonic fibroblasts. **A**, Oil red O staining of primary mouse embryonic fibroblasts from *Plac8^+/+^*; *Plac8^+/−^* and *Plac8^−/−^* mice 7 days after induction of differentiation. Upper row: culture dishes; lower row: photomicrographs of the cells (scale bars = 50 µm). **B**, mRNA expression of *aP2*, *PPARγ2 and adiponectin* at day 0 and day 7 after induction of differentiation. One representative experiment out of three is shown. Values are means ± SD. *p<0.05 *vs. Plac8^+/+^* at day 7, #p<0.05 *vs. Plac8^+/+^* at day 0.

As another model to assess the requirement for *Plac8* to induce adipogenesis, we established embryonic fibroblasts (MEF) from control, *Plac8^+/−^* and *Plac8^−/−^* mice. Differentiation of wild-type MEF into ORO positive cells was readily achieved with the differentiation cocktail. Differentiation of the heterozygous mutant MEF was less efficient and almost undetectable in the knockout MEF (Figure 3A), as also revealed by measuring expression of *aP2*, *PPARγ2* and *adiponectin*. We attempted to rescue differentiation of *Plac8* knockout MEF by retroviral transduction of *Plac8*. However, in the absence of *Plac8*, MEF proliferate extremely slowly, in agreement with a previous report showing that knockdown of Plac8 markedly reduces cell proliferation rate [16]. This decreased proliferation rate prevented retrovirus-mediated gene transduction.

**Figure 4:**
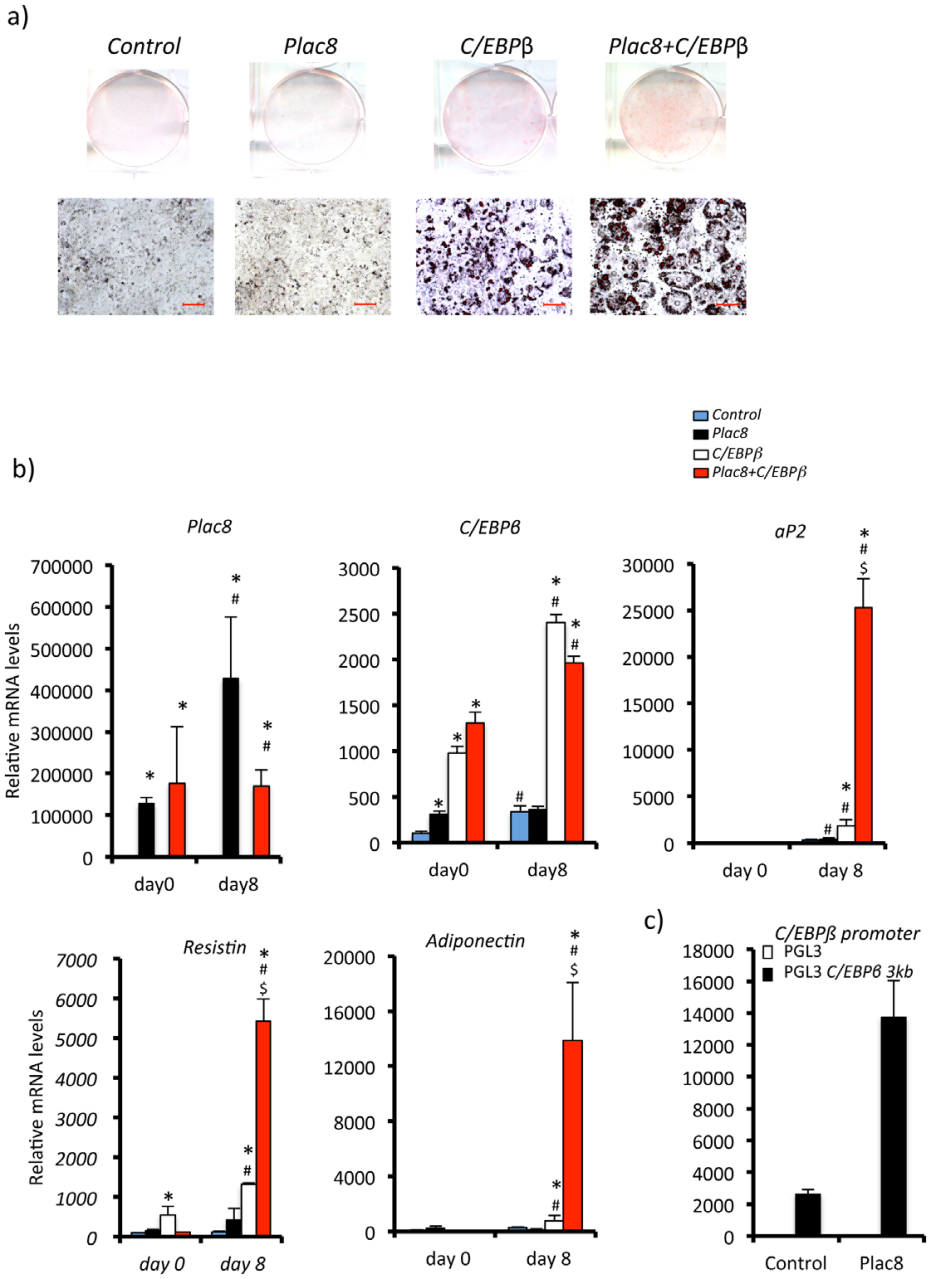
*Plac8* increases *C/EBPβ*-induced adipogenesis of NIH-3T3 fibroblasts. **A**, NIH-3T3 cells were transduced with *Plac8* and *C/EBPβ*, or control retroviruses and Oil red O staining was performed 7 days after induction of differentiation. Upper row: culture dishes; lower row: photomicrographs of the cells (scale bars = 50 µm). **B**, mRNA levels of the *Plac8*, *C/EBPβ* and *aP2* and of the white fat genes *adiponectin* and *resistin* at days 0 and 7 after induction of differentiation. One representative experiment out of three is shown. Values are means ± SD, n = 4. *p<0.05 *vs. Plac8^+/+^* at day 7, #p<0.05 *vs. Plac8^+/+^* at day 0, $p<0.05 vs. *Plac8^−/−^* at day 7. **C**, *Plac8* transactivates *C/EBPβ* and *Krox20* promoters in 3T3-L1 preadipocytes. Transcriptional activity of a 3 kb *C/EBPβ* promoter-luciferase reporter construct (black bars) or empty vector (pGL3) (white bars, close to the zero level and not visible) cotransfected into 3T3-L1 preadipocytes with a *Plac8* expression or an empty vector. Luciferase activities were determined 48 hours after transfection. Results are means ± SD (n = 3), one representative experiment is shown. *p<0.05 *vs.* empty vector.

To get another independent assessment of the role of *Plac8* in white adipogenesis, we transduced NIH 3T3 cells with *C/EBPβ*, which is known to allow adipocyte differentiation in the presence of rosiglitazone. Figure 4 shows that *Plac8* by itself did not induce significant ORO accumulation (Figure 4A) but is an inducer of *C/EBPβ* (Figure 4B). However, when transduced together with *C/EBPβ* it led to a marked increase in ORO staining and expression of *aP2*, *resistin*, and *adiponectin* as compared to *C/EBPβ* transduction alone (Figure 4A,B).

The above data therefore indicated that *Plac8* was required for white adipogenesis in 3T3-L1 cells, MEF, and NIH 3T3 cells. They also suggested that *Plac8* was an inducer of *C/EBPβ* expression. This was directly tested by *C/EBPβ*-promoter reporter assays in transfected 3T3-L1 adipoyctes. As shown in Figure 4C, co-transfection of *Plac8* with the *C/EBPβ* reporter construct markedly increased luciferase activity of the reporter construct.

**Figure 5:**
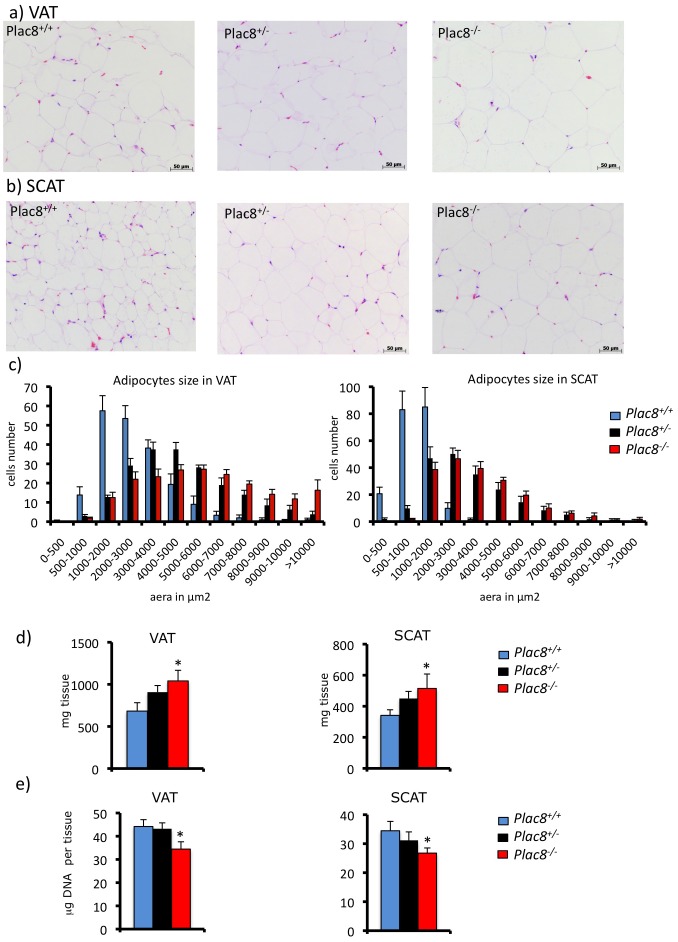
Adipocytes in visceral and subcutaneous adipose tissues are enlarged in *Plac8^−/−^* mice. Hematoxylin-eosin staining of VAT (**A**) and SCAT (**B**) sections of 29-weeks-old *Plac8^+/+^*, *Plac8^+/−^* and *Plac8^−/−^* mice (scale bars = 50 µm). **C**, Analysis of VAT and SCAT adipocytes size distribution of *Plac8^+/+^*, *Plac8^+/−^* and *Plac8^−/−^* mice. **D**, VAT and SCAT weight in *Plac8^+/+^*, *Plac8^+/−^* and *Plac8^−/−^* mice. **E**, Total DNA content in VAT and SCAT of the same mice. Values are means ± SEM (n = 7–13). *p<0.05 *vs. Plac8^+/+^*.

Mice with genetic inactivation of *Plac8* develop late-onset obesity [11]. To assess whether the obesity phenotype was associated with defect in white adipocytes, we first performed histological analysis of visceral and subcutaneous white fat depots. Figure 5A, B, C shows that white adipocytes from both fat depots from heterozygous and homozygous *Plac8* knockout mice had increased size. Weight measurements showed increased visceral and subcutaneous fat mass in heterozygous and homozygous knockout mice (Figure 5D). Importantly, however, the total DNA content of these fat depots was lower in the *Plac8* knockout mice indicating that even though their mass was greater, the number of cells forming these tissues was smaller (Figure 5E).

**Figure 6:**
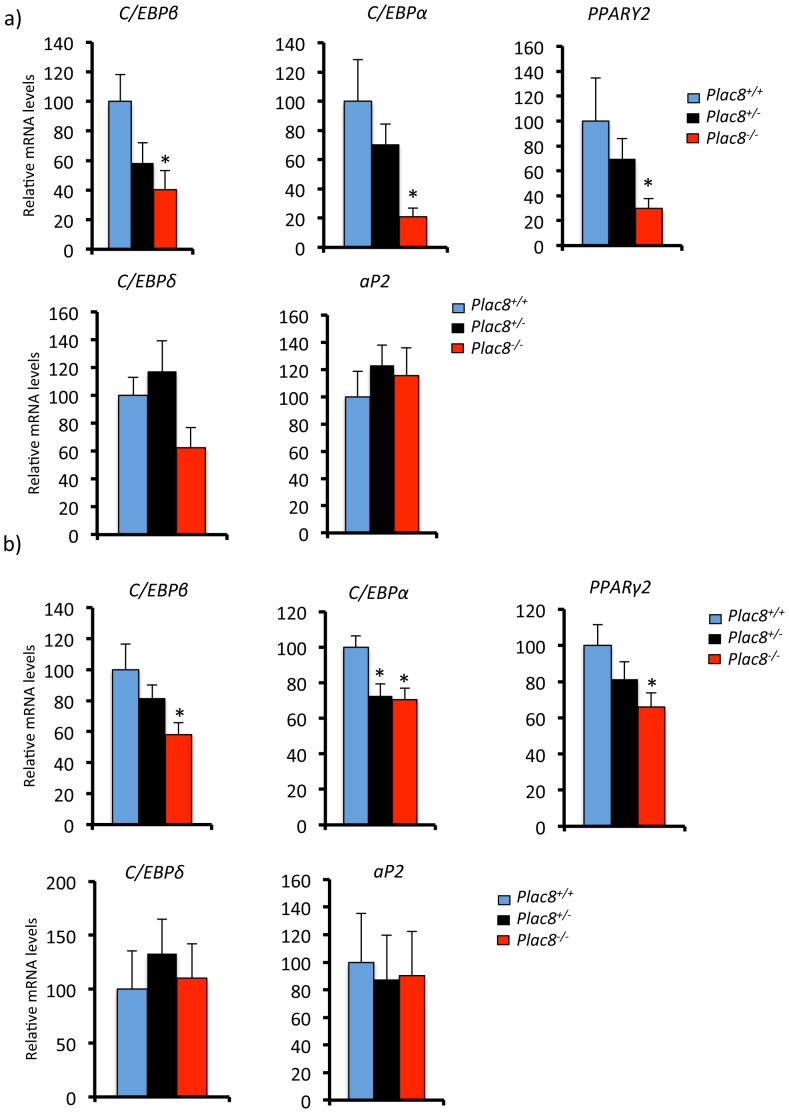
Adipogenic transcriptional regulators are decreased in white adipose tissues of *Plac8^−/−^* mice. mRNA levels of the transcriptional regulators *C/EBPβ*, *C/EBPα*, *PPARγ2*, *C/EBPδ* and of *aP2* in VAT (**A**) and SCAT (**B**) of 24 weeks-old *Plac8^+/+^*, *Plac8^+/−^* and *Plac8^−/−^* mice. Values are means ± SEM (n = 7–13), *p<0.05 *vs. Plac8^+/+^*.

**Figure 7:**
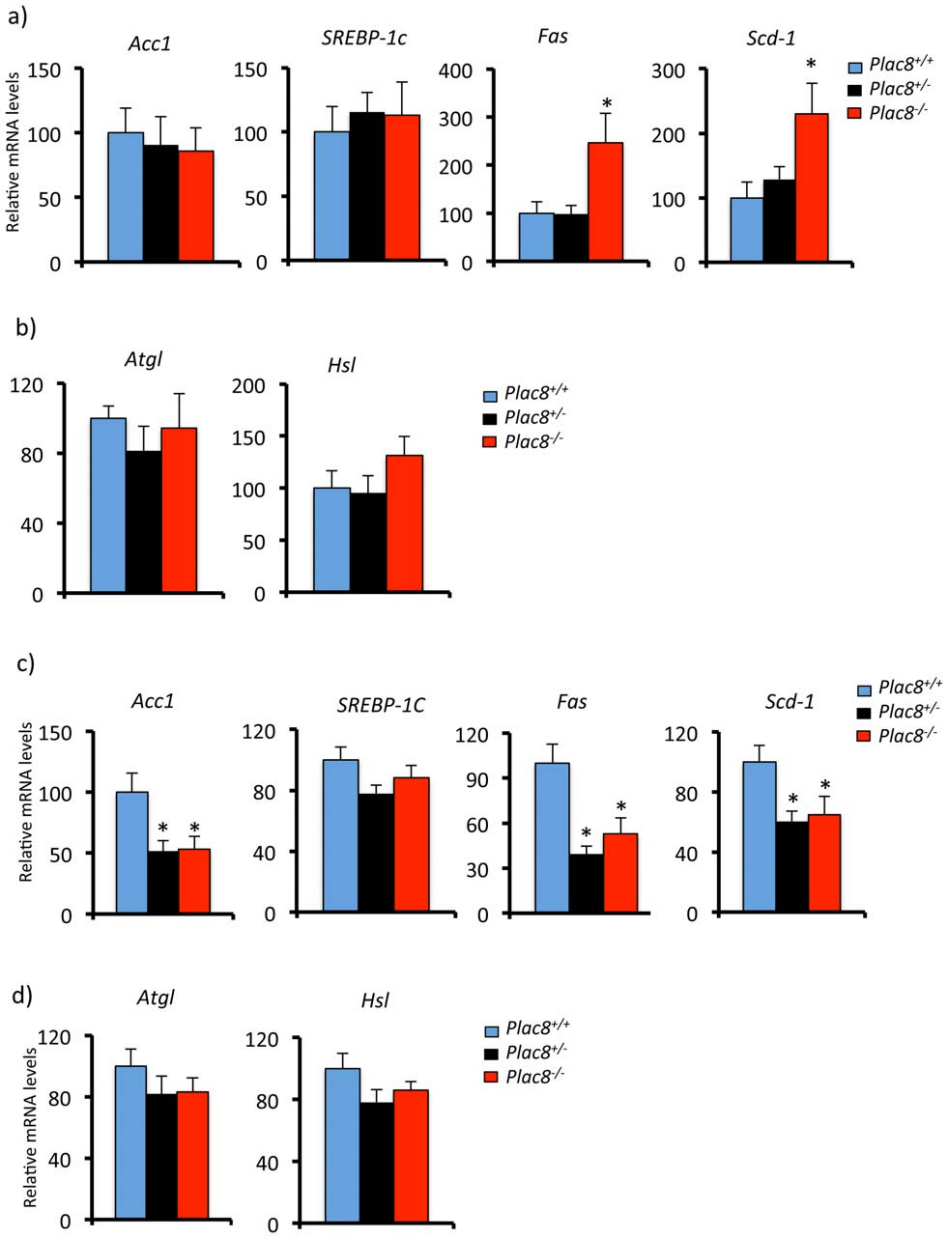
Expression of lipogenic and lipolytic genes in VAT and SCAT of *Plac8^−/−^* mice. mRNA levels of the lipogenic genes *Acc1*, *SREBP-1c*, *Fas and SCD-1*, and of the lipolytic genes *Atgl and Hsl* in VAT (**A,B**) and (**C,D**) of 24 weeks-old *Plac8^+/+^*, *Plac8^+/−^* and *Plac8^−/−^* mice. Values are means ± SEM (n = 7–13), *p<0.05 *vs. Plac8^+/+^*.

Gene expression analysis revealed that this was associated with reduced expression of *C/EBPβ*, *C/EBPα*, and *PPARγ2* in both VAT and SCAT but no significant reduction of *C/EBPδ* and *aP2* (Figure 6A,B). These data therefore indicated that absence of *Plac8* led to reduced total number of white adipocytes but enlargement of the existing ones and reduced expression of critical adipogenic transcription factors. Analysis of lipolytic and lipogenic gene expression showed that, in visceral adipose tissue, the levels of *Acc1* and *Srebp-1c* were normal but those of *Fas* and *Scd-1* were increased in *Plac8^−/−^* but not *Plac8^+/−^* adipose tissue (Figure 7A). Expression of the lipolytic genes *Atgl* and *Hsl* were, however, not different between geneotypyes (Figure 7B). In subcutaneous fat, there was no change in *srebp-1c* expression but reduced expression of *Acc1*, *Fas* and *Scd-1* and no change in *Atgl* and *Hsl* (Figure 7C,D). Expression of the inflammatory cytokines TNFα and Il-6 was not increased in the adipose tissue of *Plac8* knockout mice (not shown).

**Figure 8:**
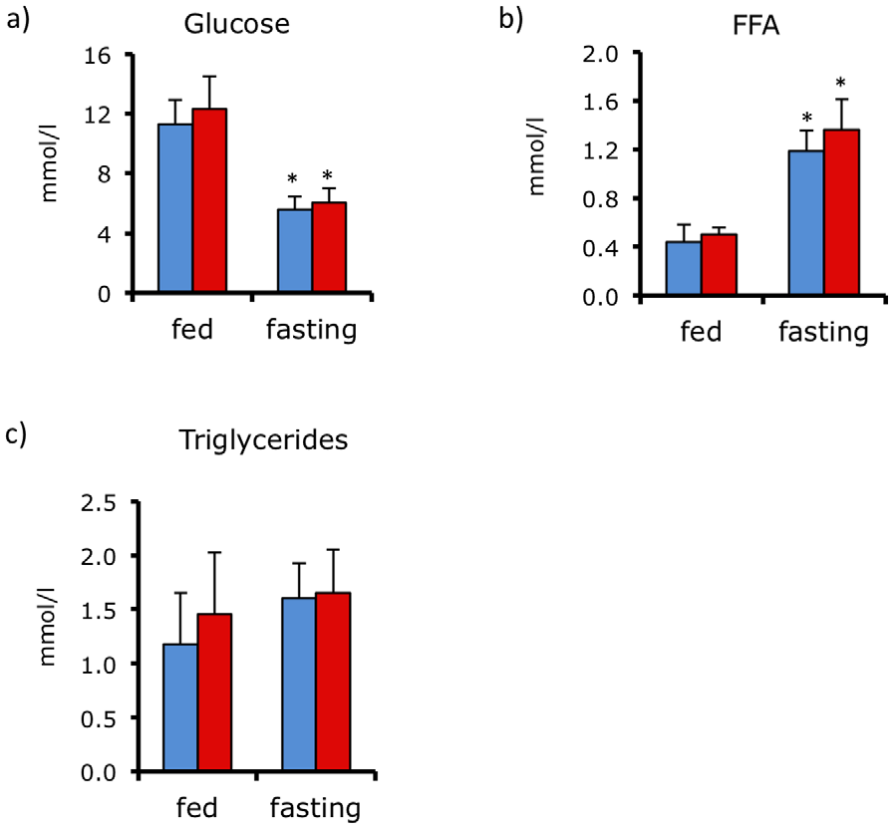
Normal glycemia and plasma free fatty acids and triglycerides in *Plac8^−/−^* mice. Blood glucose (**A**), and plasma free fatty acids (FFA) (**B**) and triglycerides (**C**) levels in fed and 16-hours fasted *Plac8^+/+^* and *Plac8^−/−^* 24-weeks old mice Values are means ± SEM (n = 7–13), *p<0.05 *vs. Plac8^+/+^* at fed state.

As defect in brown fat function and obesity are often associated with abnormal glucose homeostasis, we measured plasma glucose, free fatty acid, and triglyceride concentrations in fed and fasted control and *Plac8^−/−^* mice. Figure 8A, B, C show that these parameters were indistinguishable between control and knockout mice.

## Discussion

This study demonstrates that *Plac8* is required for in vitro white fat adipogenesis. In vivo, however, *Plac8* is dispensable for the formation of white adipocytes but its absence leads to obesity with apparently fewer but greatly enlarged adipocytes. The defect in thermoregulation and increased adiposity are, however, not associated with changes in glucose homeostasis nor in plasma free fatty acid and triglyceride levels.

*Plac8* is expressed in both white and brown fat and in both tissues it is enriched in the stromal-vascular fraction containing preadipocytes. General *Plac8* inactivation in mice leads to a phenotype of cold intolerance that can be explained by impaired fat oxidation and thermogenesis by brown adipose tissue [11]. The late-onset obesity may, however, result from combined defects in both brown and white fat differentiation and function. Here, our studies aimed to determine the role of *Plac8* in white adipogenesis and in white fat in *Plac8^−/−^* mice.

In vitro, absence of *Plac8* expression prevented differentiation into adipocytes of 3T3-L1 preadipocytes, of NIH 3T3 cells, and of MEF derived from *Plac8^−/−^* mice. Kinetics analysis of transcription factor expression upon induction of 3T3-L1 adipocytes differentiation revealed that *Plac8* expression peaked at ∼3 hours, a kinetics similar to that of *Krox20* and *C/EBPβ* expression. Analysis of *Krox20*, *Klf4*, and *C/EBPβ* expression in 3T3-L1 cells in which *Plac8* was knocked down showed that their expression was reduced. In vivo, *Plac8^−/−^* visceral and subcutaneous fat tissues showed reduced expression of *C/EBPβ*, *C/EBPα* and *PPARγ2*. These data therefore suggested that *Plac8* is an upstream regulator of the adipogenic transcription cascade. This is in agreement with our studies in BAT preadipocytes, which showed that *Plac8* is an upstream regulator of *C/EBPβ* transcription [11]. This was also confirmed by demonstrating that *Plac8* activated the transcriptional activity of a *C/EBPβ* promoter reporter construct expressed in 3T3-L1 adipocytes. Quantitative RT-PCR analysis also showed that absence of *Plac8* led to a reduced expression of *Klf4* and *Krox20*, two factors required early for induction of adipogenesis [7], [8] and which acts, at least in part, through induction of C/EBPβ. Thus, *Plac8* appears as an upstream regulator of adipogenesis, acting rapidly after induction of differentiation to trigger the adipogenic transcription cascade.

In our present studies differentiation of 3T3-L1 and NIH3T3 cells could be increased by *Plac8* transduction, which ensures a stable protein expression for periods of time that are much longer than the transient induction seen upon induction of preadipocyte differentiation. Because Plac8 has been reported to increase the proliferation of certain cell types [16], [17] and that adipogenic differentiation requires growth-arrest, the adipocyte differentiation we observed here may underestimate of the role of Plac8 since it can induce two apparently opposed cellular effects. On the other hand, the adipogenic function of Plac8 depends on its transient interaction with C/EBPβ, an event that immediately follows the induction of differentiation, possibly as a result of posttranslational modifications induced by the differentiation cocktail [11]. So far, there is no information about Plac8 posttranslational modifications, or its interaction with posttranslational modifications of other proteins. This information is required to fully understand how Plac8 contributes to increased adipogenesis.

Plac8 is a relatively small protein consisting of 124 amino acids containing an evolutionarily-conserved cysteine-rich domain that directs interaction with other proteins. Because Plac8 does not have any structural motif usually associated with transcription factors and due to its small size, its function in adipogenesis is probably to regulate the cellular localization, function, or stability of other proteins, such as through its binding to C/EBPβ, which is required for the C/EBPβ-Plac8 complex to bind to the *C/EBPβ* promoter to induce this gene transcription [11].

In vivo, *Plac8* is not absolutely required for either brown or white fat differentiation since both tissues are present in the *Plac8^−/−^* mice. This observation is analogous to that found, for instance with *C/EBPβ* or *Klf15*. Suppressed activity of either gene in 3T3-L1 fibroblasts prevents their adipogenic differentiation but knockout of either gene in mice does not prevent appearance of white fat depots [9], [18], [19], [20]. In *Plac8^−/−^* mice absence of *Plac8* leads to a decreased expression of important adipogenic genes such as *C/EBPβ*, *C/EBPα* and *PPARγ2* but not of other genes such as *C/EBPδ* and *aP2*. This, however, does not prevent augmented fat storage in this tissue. Obesity is usually associated with increase in both adipose cell number and cell size [21], [22], [23]. Interestingly, in *Plac8^−/−^* mice obesity is not associated with increased cell number but our data rather suggest a decrease in total adipocyte number. This suggests, that even though adipogenesis during development may proceed normally, the recruitment of new adipocytes from preadipocytes during the adult age may be defective even though the fat storage capacity of existing adipocytes is preserved. This fat cell enlargement occurs in the absence of changes in lipolytic gene expression and with an opposite regulation of lipogenic gene expression, which were increased in VAT (*fas* and *scd-1*) whereas these genes and *acc-1* were decreased in SCAT. The meaning of this opposite regulation is not yet clear.

Decreased BAT activity and increased white fat mass are thought to favor deregulation of glucose homeostasis. However, in the normal chow fed, 29 weeks-old control, *Plac^+/−^ Plac8^−/−^* mice studied here fed and fasted glycemia as well as plasma free fatty acids and triglycerides were indistinguishable. This, therefore, suggest that in the conditions studied the defects in thermoregulation and increased white fat mass were not causing major deregulation of glucose homeostasis. We cannot, however, exclude the presence of subtle deregulations in glucose or lipid metabolism, which would require more detailed analysis.

In summary, our data show that *Plac8* is required for in vitro adipogenesis and functions upstream of *C/EBPβ* and possibly *Krox20* and *Klf4* in triggering adipogenesis. In vivo, *Plac8* is dispensable for the production of WAT and BAT. However, because there are fewer white adipocytes in *Plac8^−/−^* mice, this suggests that the recruitment of new adipocytes during adult life may be impaired and that the phenotype of the *Plac8^−/−^* mice depends on complex interactions between defects in both white and brown adipose tissues.

## Supporting Information

Figure S1 **Plac8 knockdown in 3T3-L1 decreases adipogenesis.** mRNA levels of the transcriptional regulators *Ebf1*, *Gata2*, *Gata3*, *Nur77* and of *Cyclin D1* during differentiation of *Plac8* shRNA1 and 2 and control transduced 3T3-L1 cells at day 0 and 7. One representative experiment out of three is shown. Values are means ± SD, n = 3,*p<0.05 *vs.* shLuc, #p<0.05 *vs.* shLuc at day 0
(TIF)

Figure S2 ***Rosiglitazone does not restore the full adipogenic phenotype in shRNA expressing 3T3-L1 cells*** **.** **A**, Oil red O staining of 3T3-L1 transduced with two different shRNA against *Plac8* or the control shRNA (shLuc) at 7 days after induction of differentiation in the presence of rosiglitazone in the differentiation cocktail. Upper row: culture dishes; lower row: photomicrographs of the cells (scale bars = 100 µm). **B**, mRNA levels *aP2* and *PPARγ2* during at day 0 and 7 of differentiation. One representative experiment out of three is shown. Values are means ± SD, n = 3.,*p<0.05 *vs.* shLuc, #p<0.05 *vs.* shLuc at day 0.
(TIF)
