# Supplementary figures and images for: Plac8 is required for White Adipocyte Differentiation in vitro and Cell Number Control in vivo
Source: PLoS One. 2012 Nov 14;7(11):e48767. doi: 10.1371/journal.pone.0048767 (PMC3498234; doi:10.1371/journal.pone.0048767)

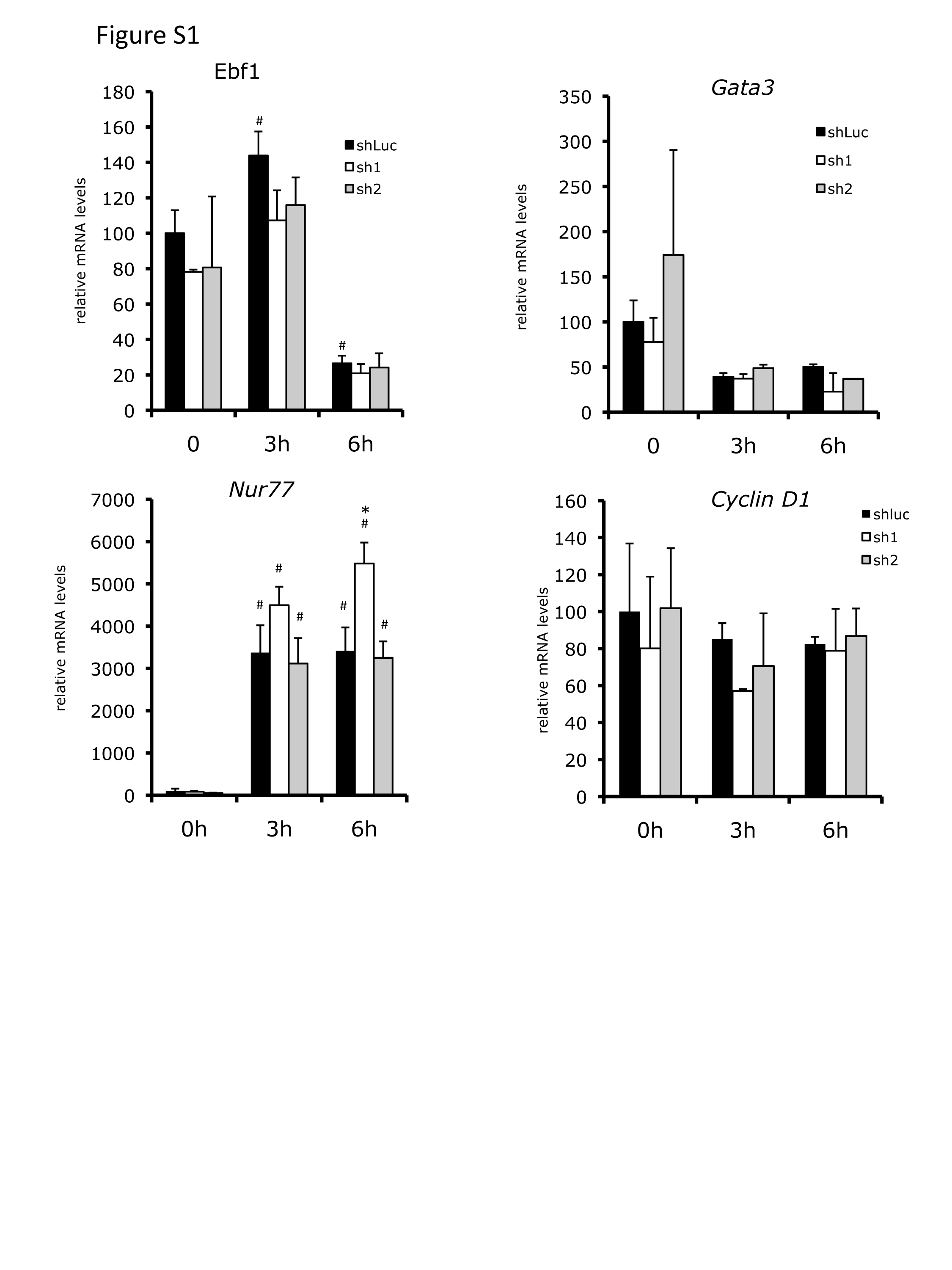

Supplement: Figure S1 — Plac8 knockdown in 3T3-L1 decreases adipogenesis. mRNA levels of the transcriptional regulators Ebf1, Gata2, Gata3, Nur77 and of Cyclin D1 during differentiation of Plac8 shRNA1 and 2 and control transduced 3T3-L1 cells at day 0 and 7. One representative experiment out of three is shown. Values are means ± SD, n = 3,*p<0.05 vs. shLuc, #p<0.05 vs. shLuc at day 0 (TIF) [file pone.0048767.s001.tif]

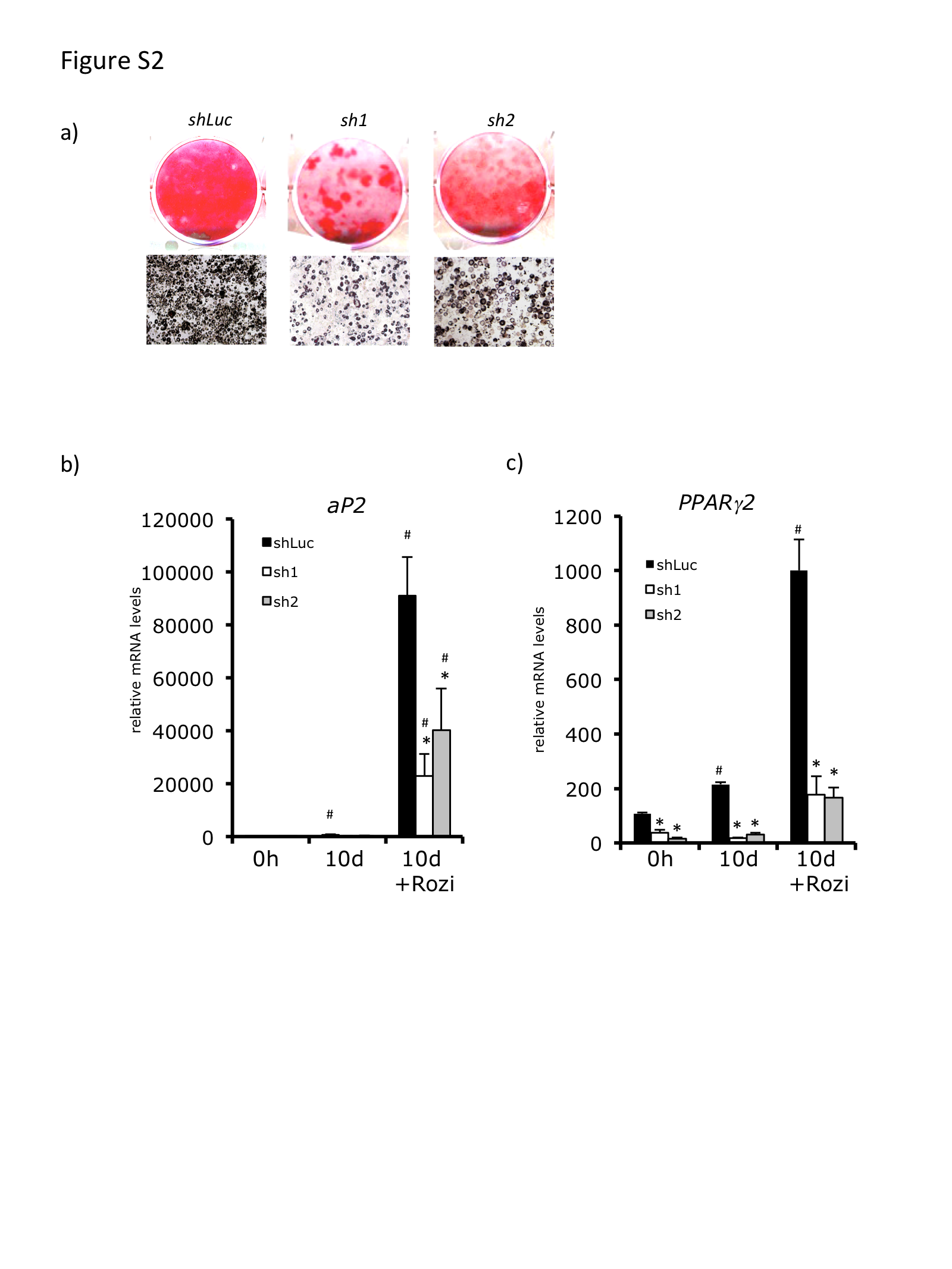

Supplement: Figure S2 — Rosiglitazone does not restore the full adipogenic phenotype in shRNA expressing 3T3-L1 cells . A, Oil red O staining of 3T3-L1 transduced with two different shRNA against Plac8 or the control shRNA (shLuc) at 7 days after induction of differentiation in the presence of rosiglitazone in the differentiation cocktail. Upper row: culture dishes; lower row: photomicrographs of the cells (scale bars = 100 µm). B, mRNA levels aP2 and PPARγ2 during at day 0 and 7 of differentiation. One representative experiment out of three is shown. Values are means ± SD, n = 3.,*p<0.05 vs. shLuc, #p<0.05 vs. shLuc at day 0. (TIF) [file pone.0048767.s002.tif]
